# A Heat-Shock Protein Axis Regulates VEGFR2 Proteolysis, Blood Vessel Development and Repair

**DOI:** 10.1371/journal.pone.0048539

**Published:** 2012-11-06

**Authors:** Alexander F. Bruns, Nadira Yuldasheva, Antony M. Latham, Leyuan Bao, Caroline Pellet-Many, Paul Frankel, Sam L. Stephen, Gareth J. Howell, Stephen B. Wheatcroft, Mark T. Kearney, Ian C. Zachary, Sreenivasan Ponnambalam

**Affiliations:** 1 Endothelial Cell Biology Unit, School for Molecular and Cellular Biology, University of Leeds, Leeds, United Kingdom; 2 Division of Cardiovascular and Diabetes Research, Faculty of Medicine and Health, University of Leeds, Leeds, United Kingdom; 3 Centre for Cardiovascular Biology and Medicine, University College London, London, United Kingdom; Bristol Heart Institute, University of Bristol, United Kingdom

## Abstract

Vascular endothelial growth factor A (VEGF-A) binds to the VEGFR2 receptor tyrosine kinase, regulating endothelial function, vascular physiology and angiogenesis. However, the mechanism underlying VEGFR2 turnover and degradation in this response is unclear. Here, we tested a role for heat-shock proteins in regulating the presentation of VEGFR2 to a degradative pathway. Pharmacological inhibition of HSP90 stimulated VEGFR2 degradation in primary endothelial cells and blocked VEGF-A-stimulated intracellular signaling via VEGFR2. HSP90 inhibition stimulated the formation of a VEGFR2-HSP70 complex. Clathrin-mediated VEGFR2 endocytosis is required for this HSP-linked degradative pathway for targeting VEGFR2 to the endosome-lysosome system. HSP90 perturbation selectively inhibited VEGF-A-stimulated human endothelial cell migration *in vitro*. A mouse femoral artery model showed that HSP90 inhibition also blocked blood vessel repair *in vivo* consistent with decreased endothelial regeneration. Depletion of either HSP70 or HSP90 caused defects in blood vessel formation in a transgenic zebrafish model. We conclude that perturbation of the HSP70-HSP90 heat-shock protein axis stimulates degradation of endothelial VEGFR2 and modulates VEGF-A-stimulated intracellular signaling, endothelial cell migration, blood vessel development and repair.

## Introduction

Vascular endothelial growth factors (VEGFs) are a family of cytokines that bind cell surface receptors and regulate key steps in both physiological and pathological angiogenesis [1], [2]. Under conditions of hypoxia, e.g. during tumor growth, synthesis of the VEGF-A isoform is increased, stimulating tumor neovascularization and enrichment of its oxygen and nutrient supply [1]. The VEGF-A gene gives rise to multiple splice variants with distinct functional properties: VEGF-A_165_ (designated as VEGF-A herein) is the most abundant and physiologically active variant, playing a central role in pathophysiological states such as cancer and macular degeneration [3]. VEGF-A binds two structurally-related receptor tyrosine kinases on vascular endothelial cells: VEGF receptor 1 (VEGFR1, Flt-1) and 2 (VEGFR2, KDR, Flk-1). Despite similarities, VEGFR2 largely mediates VEGF-A-induced pro-angiogenic signaling whereas VEGFR1 acts as a ‘decoy’ receptor that sequesters VEGF-A. Ligand binding to VEGFR2 promotes receptor dimerization and tyrosine kinase activation. This stimulates downstream signaling including activation of the c-Raf/MEK/ERK and PI3K/Akt pathways leading to increased cell proliferation, migration and survival [2].

A family of benzoquinone ansamycins that have weak antibiotic activity include the potential anti-cancer drug geldanamycin [4]. This compound and related analogs are inhibitors of the heat-shock protein of 90 kDa (HSP90) and activate a degradative pathway involving the cytosolic 26S proteasome [5]–[7]. ‘Client’ proteins recognized by this pathway include ErbB2, eNOS, Akt and mutant p53 [4]–[6]. Geldanamycin stimulates proteolysis of the ErbB2 receptor tyrosine kinase via endosome-lysosome trafficking, thus modulating epithelial cell proliferation, tumor progression and metastasis [8], [9]. Geldanamycin has been implicated previously as an inhibitor of tumor angiogenesis [10], [11]. Endothelial VEGFR2 is a receptor tyrosine kinase which is a key regulator of vasculogenesis and angiogenesis [12]. Ligand-stimulated VEGFR2 undergoes proteolysis involving lysosome- and proteasome-linked activities [13], [14] raising the possibility that heat-shock proteins (HSPs) regulate this process. In this study, we investigated the role played by HSPs in regulating the stability and turnover of VEGFR2 and subsequent VEGF-A-regulated responses: intracellular signaling, endothelial cell migration and blood vessel repair.

## Methods

### Ethics Statement

Human umbilical cords used for isolation and culture of primary endothelial cells were provided by written informed consent and under ethical approval (reference CA03/020) by the Leeds NHS Hospitals Local Ethics Committee (UK). Mouse and zebrafish studies were carried carried out in accordance with local and national regulations under an animal project licence approved by the UK Home Office.

### Materials and Cell Culture

Primary human umbilical vein endothelial cells (HUVECs) were isolated and prepared as previously described [14], [15]. The geldanamycin analog 17-(allylamino)-17-demethoxygeldanamycin (17-DMAG) was from LC Labs (Woburn, USA) and radicicol was from Alomone Labs (Jerusalem, Israel). Recombinant cytokines used included VEGF-A (Genentech Inc., San Francisco, USA) and basic FGF (R&D Systems, Minneapolis, USA). The lentiviral expression plasmid pSINCSHWδ/*Not*I was a gift from Y. Ikeda (Mayo Clinic, Minnesota, USA). Endothelial cell growth medium was from PromoCell (Heidelberg, Germany). All other materials and reagents were from Sigma-Aldrich (Poole, UK) unless otherwise stated.

### Immunoblotting and Quantification

HUVEC lysate preparation and immunoblot analysis were performed as described previously [13], [16]. The following antibodies were used in the present study: anti-VEGFR1, anti-VEGFR2 (R&D Systems), anti-Akt, anti-phospho-Akt (Ser^473^), anti-ERK1/2, anti-phospho-ERK1/2 (Thr^202^/Tyr^204^), anti-phospho-VEGFR2 (Tyr^1175^), anti-phospho-PLCγ1 (Tyr^783^), anti-CHIP (Cell Signaling Technology), anti-α-tubulin (Sigma-Aldrich), anti-HSP90α, anti-HSP90β, anti-HSP70 (Enzo Life Sciences, Exeter, UK), anti-HSC70, anti-transferrin receptor (TfR), anti-PLCγ1 (Santa Cruz Biotechnology, USA), anti-FLAG M2 (Sigma-Aldrich), anti-GFP (Acris Antibodies, Germany) and X22 monoclonal anti-clathrin heavy chain (from A. Jackson, Cambridge, UK). Immunoreactive bands were visualised using an enhanced chemiluminescence detection kit (Geneflow, Nottingham, UK). Anti-α-tubulin or anti-transferrin receptor antibodies were used as an internal control for normalizing the loaded samples.

### Immunoprecipitation and Cell Surface Biotinylation

Immunoprecipitations were performed for 2 h at 4°C using protein G-Sepharose (Millipore, Durham, UK), 0.5 µg of antibody and 500 µg of total protein in lysis buffer (0.5% (w/v) digitonin, 100 mM KCl, 20 mM Hepes pH 7.4, 80 mM sucrose, 1 mM MgCl_2_, 10 mM potassium acetate, 1 mM sodium vanadate, 50 mM sodium fluoride, and protease inhibitors). Samples (25 µg of protein or total bead volume) were analyzed by SDS-PAGE and immunoblotting as previously described. For analysis of cell surface protein levels, cells were treated as appropriate and labeled with EZ-Link Sulfo NHS-LC biotin (ThermoFisher Scientific) in PBS containing divalent cations on ice for 45 min with gentle agitation. Cells were washed and lysed as above followed by immunoprecipitation of VEGFR2 and transferrin receptor (control) as described above. Beads were washed thoroughly in lysis buffer and proteins eluted in SDS-PAGE sample buffer prior to electrophoresis alongside whole cell lysate samples and immunoblotting using streptavidin-HRP.

### RNA Interference (RNAi )

HUVECs were transfected with either no siRNA (small interfering RNA) (control), 10 nM control siRNA (mock; 5′-UAGCGACUAAACACAUCAA-3′), or 10 nM annealed HSP70 ‘a’ siRNA (5′-UGACGAAAGACAACAAUCU-3′), HSP70 ‘b’ siRNA (5′-CCAAGGUGCAGGUGAGCUA-3′), CHC17 siRNA (5′-GGGUGCCAGAUUAUCAAUU-3′) or CHIP siRNA (5′-CGCAUUCAUCUCUGAGAAU-3′) using RNAiMAX transfection reagent (Invitrogen, Amsterdam, Netherlands) according to the manufacturer’s instructions. Cells were recovered for 48–72 h prior to lysis and immunoblot analysis as described previously [13], [17].

### Lentiviral Transduction

For protein overexpression human HSP70-FLAG was generated by PCR and subcloned into the lentiviral expression plasmid pSINCSHWδ/*Not*I. Lentiviral particles carrying HSP70-FLAG were generated using previously described protocols [18]. Recombinant lentiviruses carrying the HSP70-FLAG transgene were used to transduce HUVECs. Cells were processed for microscopy 48 h after viral transduction.

### Cell Migration Assay

For cell migration assay, HUVECs were trypsinized and seeded at 5×10^4^ cells per ml into a 24-well plate with 8 µm pore size Transwell inserts (Becton-Dickinson, Oxford, UK) with the specified concentration of geldanamycin in the upper chamber and 25 ng/ml of recombinant VEGF-A or basic FGF in the lower chamber [13]. After 16 h, filters were fixed, stained with hematoxylin-eosin and excised for microscopy. Random fields from each image were counted for calculation of % number of cells migrated onto filter underside versus non-treated control.

### Immunofluorescence Microscopy

Cells were processed as described previously [14]. Endogenous VEGFR2 and overexpressed HSP70-FLAG were detected with antibodies against VEGFR2 and the FLAG-epitope respectively and visualized with AlexaFluor488- and AlexaFluor594-conjugated antibodies respectively. DAPI (4′,6′-diamidino-2-pheylindole) was used to visualise nuclear DNA. Images were captured using a confocal microscope. Quantification of co-localized pixels were performed using NIH Image J.

### Mouse Femoral Artery Injury Model

C57Bl/6 mice were injected daily with 0.1 ml of control (DMSO vehicle) or geldanamycin (5 mg/kg) for 1 week. During this time, mice (n = 5 per group) were subjected to femoral artery injury operation and closure using previously described procedures [19]. After 5 days the animals were sacrificed, femoral arteries removed by dissection and subjected to Evans Blue staining. Dye penetration and staining of the femoral arteries were assessed using Image ProPlus (Media Cybernetics, Bethesda, USA). Endothelial regeneration in the injured femoral artery was compared to control uninjured femoral artery derived from the other limb.

### Transgenic Zebrafish Manipulation and Analysis

Transgenic *Fli1-GFP* zebrafish embryos were injected with:

HSP70 morpholino: 5′TAGCGATTCCTTTTGGAGAAGACAT3′; Hsp90βmorpholino: 5′CTTCTTGGCGCATTTCTTCAGGCAT3′ or control morpholino: 5′CCTCTTACCTCAGTTACAATTTATA 3′ (Gene Tools, Philomath, USA) and left to develop for 48 h. 24 h after the injections, the embryos were transferred to fish water containing 1-phenyl 2-thiourea (PTU) in order to improve signal detection by confocal microscopy and expression of GFP. Embryos were fixed overnight in 4% (w/v) PFA at 4°C and then transferred to PBS. Pictures of the zebrafish embryos were obtained under bright-field using a NIKON SMZ1500 stereomicroscope (Nikon, Kingston-Upon-Thames, UK). Embryos were stained with a rabbit anti-GFP antibody to visualize the vascular system. For immunoblotting analysis, yolk sacs were removed, lysed and sonicated in RIPA buffer (30 mM Tris-HCl, pH 7.4, 150 mM NaCl, 1% (v/v) NP-40, 0.5% (w/v) deoxycholate, 2 mM ETDA plus protease inhibitors), subjected to SDS-PAGE and antibody staining.

### Statistical Analysis

Statistical analysis was performed using either one-way analysis of variance (ANOVA) followed by Tukey’s *post hoc*-test analysis for multiple comparisons or Student’s t-test using GraphPad Prism software. Statistical significances are provided in further detail in figure legends. Unless otherwise stated in figure legends, three independent experiments were used for quantification.

## Results

### HSP90 Activity is Required for VEGFR2 Stability and Intracellular Signaling

**Figure 1:**
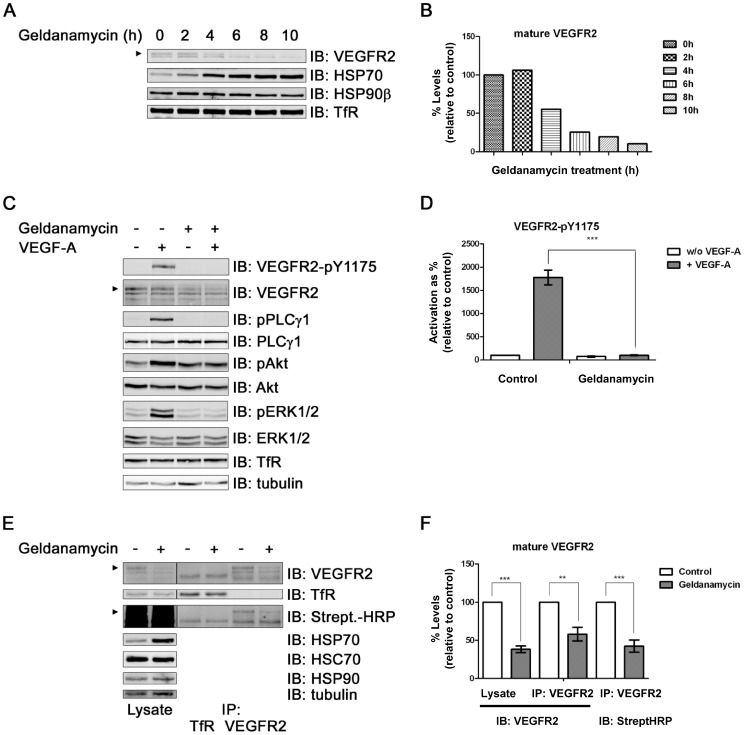
HSP90 inhibition stimulates mature VEGFR2 degradation and blocks intracellular signaling in primary endothelial cells. (A) HUVECs were subjected to geldanamycin (17-DMAG) treatment for indicated times, lysed and processed for immunoblotting (IB) to analyze indicated protein levels. Arrowhead indicates mature VEGFR2; TfR, transferrin receptor. (B) Quantification of mature VEGFR2 levels in control and geldanamycin-treated endothelial cells; data shown is representative of 3 independent experiments. (C) HUVECs were treated with dimethyl sulfoxide (DMSO; control) or geldanamycin (4 h) and stimulated with VEGF-A (5 min) followed by immunoblotting (IB) of phosphorylated and total levels of intracellular signaling enzymes. PLCγ1, phospholipase Cγ1; ERK1/2, extracellular signal-regulated kinase 1/2. (D) Immunoblot data from panel C were quantified for phosphorylated and activated VEGFR2-pY1175; error bars denote ±SEM (n≥3), ***p<0.005 using one-way ANOVA. (E) HUVECs were treated with geldanamycin (4 h) and the cell surface labeled with biotin on ice. VEGFR2 and transferrin receptor (TfR) were immunoprecipitated (IP) and analyzed by immunoblotting (IB) alongside whole cell lysates using streptavidin-HRP (Strept HRP; streptavidin-horsereadish peroxidase). HSC70, heat shock cognate protein chaperone. (F) Immunoblot data from panel E were quantified for mature VEGFR2; error bars denote ±SEM (n≥3), **p<0.01; ***p<0.005 using one-way ANOVA.

Endothelial VEGFR2 is subject to ubiquitination and degradation but the exact mechanism is unclear [13], [20]–[22]. It has been reported that heat-shock proteins are implicated in regulating VEGFR2 function and downstream signalling [23]–[27]. To test whether heat-shock protein inhibition affected VEGFR2 status, we used a geldanamycin analog to block HSP90 activity in primary human endothelial cells (Figure 1). HSP90 inhibition caused a time-dependent decrease in VEGFR2 levels (Figure 1A). Although HSP90β levels were relatively unaffected, HSP70 levels were elevated in a time-dependent manner (Figure 1A). Prolonged inhibition of HSP90 activity for 10 h showed a ∼90% decrease in VEGFR2 levels (Figure 1B). A structurally unrelated HSP90 inhibitor, radicicol, stimulated degradation of endothelial VEGFR2 in a similar manner (Figure S1A).

To test whether HSP90 inhibition affected VEGFR2-regulated intracellular signaling, we combined HSP90 inhibition for 4 h with a brief 5 min pulse with VEGF-A (Figure 1C). Ligand binding stimulated VEGFR2 activation and phosphorylation of residue Y1175 but this was completely blocked by HSP90 inhibition (Figure 1C, 1D). Importantly, quantification of downstream signaling events including phospholipase Cγ1 (PLCγ1) and ERK1/2 phosphorylation revealed >10-fold reduction in phospho-PLCγ1 and phospho-ERK1/2 levels (Figure S1B and S1C). In contrast, the levels of transferrin receptor were not significantly affected by either HSP90 inhibition or VEGF-A stimulation (Figure S1D). Given these effects on signal transduction, we hypothesized that HSP90 activity regulates mature VEGFR2 levels at the plasma membrane. To address this point we used a cell surface biotinylation approach in HUVECs (Figure 1E). HSP90 was inhibited using geldanamycin followed by biotinylation of the cell surface and immunoisolation of VEGFR2 or transferrin receptor (TfR) (see Methods). The cell surface VEGFR2 pool was analyzed by staining with streptavidin-HRP, a high-affinity binding partner of biotin (Figure 1E). Quantification of streptavidin-HRP levels showed that HSP90 inhibition stimulated ∼2-fold decrease in mature VEGFR2 levels at the plasma membrane (Figure 1F). Immunoblotting for VEGFR2 was also used to confirm a reduction in total VEGFR2 levels during geldanamycin treatment (Figure 1E). Levels of a TfR control were unaltered by the same treatment (Figure 1E).

### HSP70 is Recruited to a VEGFR2 Complex to Modulate Receptor Trafficking and Proteolysis

**Figure 2:**
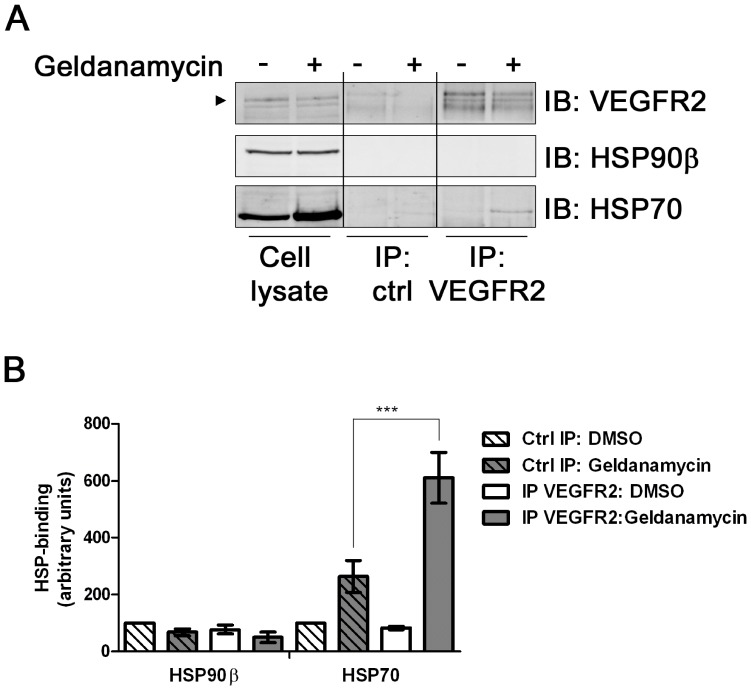
Inhibition of HSP90 stimulates HSP70-VEGFR2 interaction. (A) HUVECs pre-treated with geldanamycin for 4 h were lysed and control protein (goat IgG) or VEGFR2 immuno-isolated complexes (IP) were analyzed by immunoblotting (IB) for HSP70 or HSP90β. Arrowhead indicates mature VEGFR2. (B) Quantification of HSP70 and HSP90β association with VEGFR2 under control conditions without (−) or with (+) geldanamycin; error bars denote ±SEM (n = 6), ***p<0.005 using one-way ANOVA.

Inhibition of HSP90 activity modulates endothelial responses [26], [27]. One hypothesis to explain this effect is that one or more HSPs bind VEGFR2 and present it to a degradative pathway. To test whether HSPs could bind VEGFR2 we lysed primary endothelial cells, immunoisolated VEGFR2 complexes and tested for the presence of HSP90β and HSP70 using immunoblotting (Figure 2A). Surprisingly, endothelial cells with inhibited HSP90 showed increased HSP70 association with VEGFR2 compared to controls (Figure 2A, 2B). Importantly, HSP90β association with VEGFR2 was negligible under either control or HSP90-inhibited conditions (Figure 2A, 2B).

**Figure 3:**
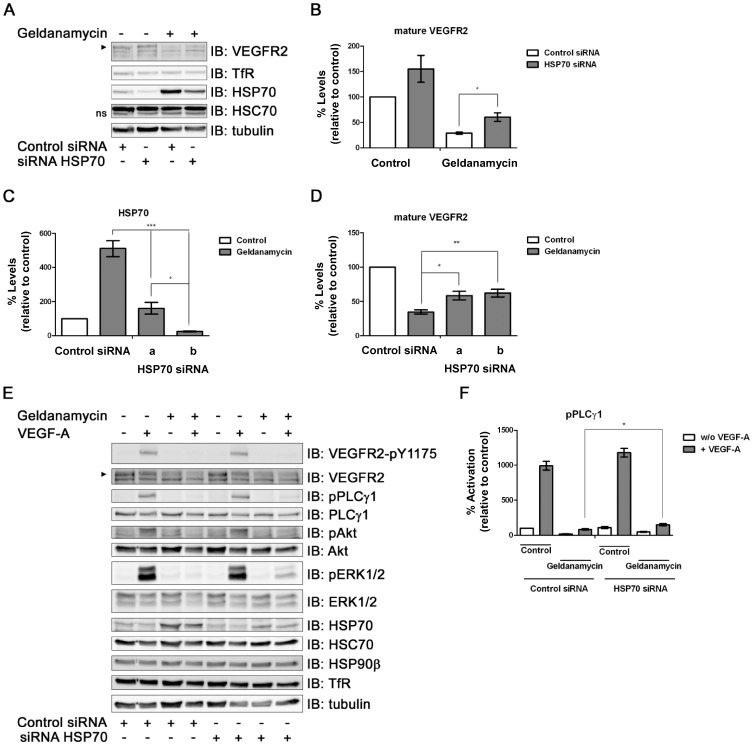
HSP70 requirement for geldanamycin-stimulated VEGFR2 degradation. (A) HUVECs pre-treated with siRNA duplex ‘a’ to HSP70 alone or with geldanamycin (4 h) were analyzed by immunoblotting (IB) for proteins as indicated. Arrowhead indicates mature VEGFR2. (B) Quantification of mature VEGFR2 levels under conditions in panel A. (C) Quantification of HSP70 levels in HUVECs pre-treated with either of two different synthetic siRNA duplexes ‘a’ or ‘b’ to HSP70±geldanamycin. (D) Quantification of VEGFR2 levels in HSP70-depleted HUVECs (duplex ‘a’ or ‘b’) ±geldanamycin (4 h). Error bars denote ±SEM (n≥3), *p<0.05; **p<0.01; ***p<0.005 using one-way ANOVA. (E) HUVECs pre-treated with siRNA duplexes for HSP70 or control siRNA were treated ±geldanamycin (4 h) ±VEGF-A (5 min) and analyzed for signaling events by immunoblotting. (F) Quantification of phospho-PLCγ1 levels from panel E. Error bars denote ±SEM (n≥3), *p<0.05 using one-way ANOVA.

To further examine a role for HSP70 in VEGFR2 down-regulation or degradation, we used two different small interfering RNA (siRNA) duplexes (‘a’ or ‘b’) to deplete HSP70 protein levels using RNA interference (Figure 3). HSP70 silencing by siRNA ‘a’ was evident in comparison to controls (Figure 3A). Intriguingly, simultaneous HSP90 inhibition and HSP70 depletion using siRNA ‘a’ caused ∼50% elevation in steady-state VEGFR2 levels compared to HSP90 inhibition alone (Figure 3B). One characteristic of inhibition of HSP90 activity is increased cellular stress response(s) which in turn elevate HSP70 levels. To consolidate this, we checked whether knockdown of HSP70 levels using either of two different siRNA duplexes ‘a’ or ‘b’ specific for HSP70 could produce similar effects on VEGFR2 stability (Figure 3C). Knockdown of HSP70 levels using either siRNA did indeed inhibit the VEGFR2 degradation caused by HSP90 inhibition (Fig. 3D), confirming this result. An important question was whether the block in VEGF-A-stimulated and VEGFR2-regulated intracellular signaling caused by HSP90 inhibition could be rescued by HSP70 depletion. Analysis of primary endothelial cells inhibited for HSP90 and stimulated with VEGF-A showed a small but significant rescue of downstream ERK1/2 phosphorylation (Figure 3E) and PLCγ1 phosphorylation (Figure 3F) upon HSP70 knockdown.

**Figure 4:**
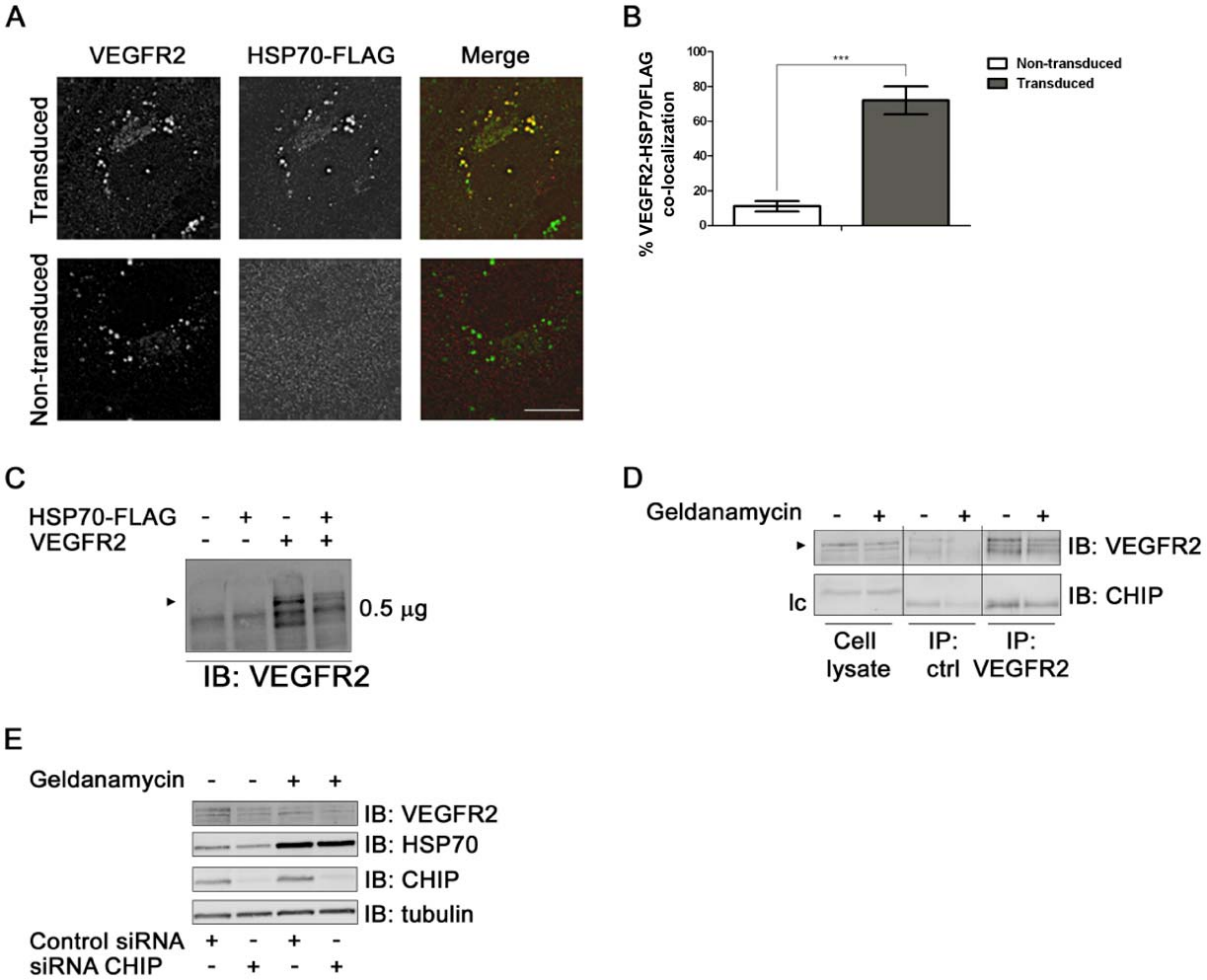
HSP70 overexpression and co-distribution with VEGFR2. (A) Lentiviral transduced HUVECs expressing human HSP70-FLAG were labeled for VEGFR2 (green) and HSP70-FLAG (red), which were detected using labeled secondary antibodies and co-distribution shown (yellow). Bar, 10 µm. (B) Quantification of VEGFR2 co-distribution with HSP70-FLAG under control or HSP70 overexpression conditions. Error bars denote ±SEM (n = 15), ***p<0.005 using Student’s t-test. (C) HEK-293T cells were transfected with VEGFR2 and/or HSP70-FLAG and processed for immunoblotting (IB) using anti-VEGFR2 antibody. Arrowhead denotes mature transfected VEGFR2. (D) HUVECs were treated ±geldanamycin (4 h) and immunoprecipitation (IP) carried out for control protein (Goat IgG) and VEGFR2 as per Figure 2. Isolated protein complexes were processed for immunoblotting (IB) using anti-VEGFR2 and anti-CHIP antibodies. Arrrowhead denotes mature VEGFR2. (E) HUVECs were treated with either control siRNA or siRNA duplex targeted against CHIP and treated ±geldanamycin (4 h). Cells were lysed and processed for imunoblotting (IB). Representative immunoblots shown. CHIP, carboxy terminus of HSP70-interacting protein.

Another approach was to assess the intracellular distribution of HSP70 relative to VEGFR2 (Figure 4). Lentiviral transduced endothelial cells revealed that HSP70-FLAG co-distributes with VEGFR2 in punctate structures resembling late endosomes or lysosomes (Figure 4A). Quantification of these microscopy data showed a high degree of overlap between the two proteins (Figure 4B). Co-expression of both human VEGFR2 and HSP70-FLAG in non-endothelial HEK-293T cells also suggested increased proteolysis (Figure 4C). A key component of the HSP90-regulated degradative pathway is the E3 ubiquitin ligase CHIP [28], [29]. This was neither present in VEGFR2 complexes (Figure 4D) nor had RNAi-mediated knockdown of CHIP any discernible effects upon mature VEGFR2 levels after HSP90 inhibition (Figure 4E).

### Clathrin-mediated Endocytosis is Required for HSP-regulated VEGFR2 Degradation

**Figure 5:**
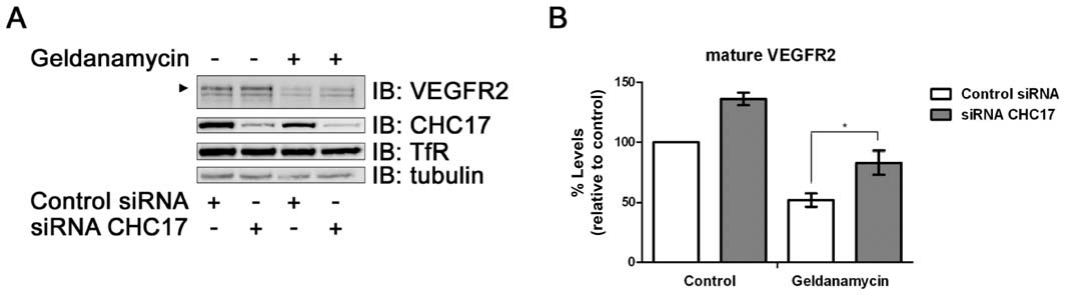
Clathrin-regulated endocytosis is a requirement for geldanamycin-stimulated VEGFR2 degradation. (A) HUVECs subjected to CHC17 knockdown were followed by geldanamycin treatment (4 h) and immunoblot analysis (IB) of indicated proteins. (B) Quantification of mature VEGFR2 levels from the experiments shown in panel A. Error bars denote ±SEM (n≥3), *p<0.05 using one-way ANOVA. CHC17, clathrin heavy chain.

Endosome-associated 26S proteasome and lysosome-mediated degradation are implicated in VEGFR2 proteolysis [13], [14]. One question was whether plasma membrane endocytosis and transport to the endosome-lysosome pathway are required for this HSP-regulated VEGFR2 degradation. To answer this, we depleted clathrin heavy chain (CHC17), a protein required for plasma membrane receptor endocytosis via clathrin-coated pits and vesicles. CHC17 knockdown caused >70% decrease in protein levels in either control or HSP90-inhibited endothelial cells (Figure 5A and S2A). In control endothelial cells, VEGFR2 levels were elevated by ∼30% upon CHC17 depletion, whereas HSP90 inhibition caused ∼50% decrease in VEGFR2 levels (Figure 5B). However, upon simultaneous HSP90 inhibition and CHC17 knockdown, VEGFR2 were levels were reduced by only ∼20% (Figure 5B), suggesting receptor-mediated endocytosis is required for this phenomenon. In contrast, transferrin receptor levels were not significantly affected by either HSP90 inhibition, CHC17 knockdown or a combination of both (Figure S2B).

### HSP90 Activity is Required for Endothelial Cell Migration, Arterial Regeneration and Blood Vessel Development

**Figure 6:**
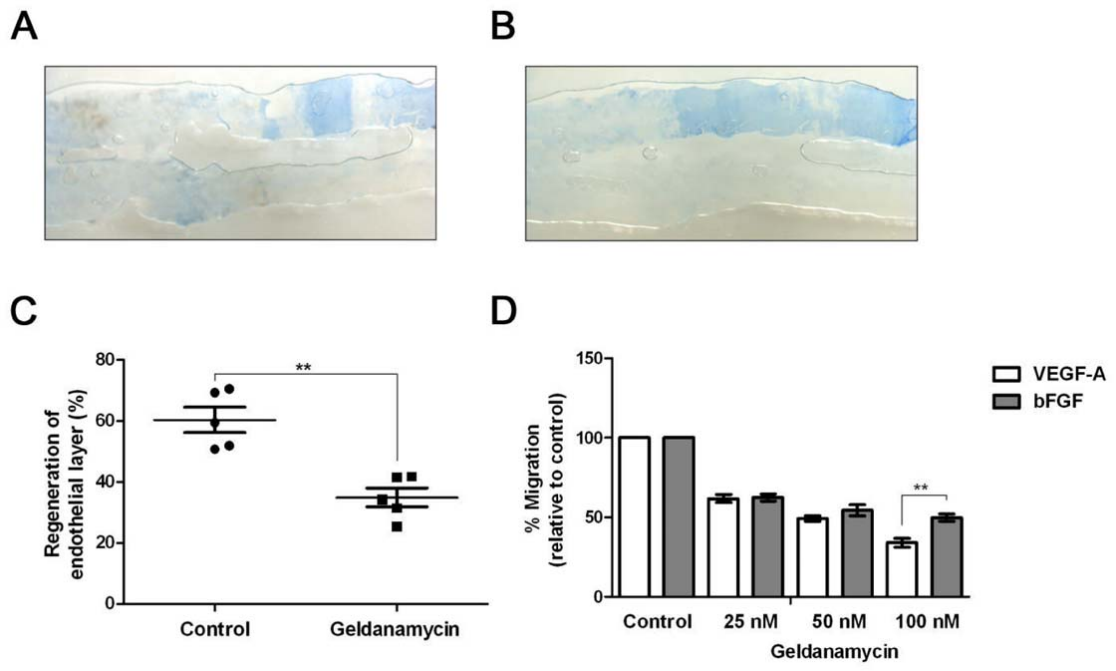
HSP90 inhibition perturbs mouse arterial repair and endothelial cell migration. (A) A representative view of femoral artery re-endothelialization in control mice (DMSO treated). (B) A representative view of femoral artery re-endothelialization in geldanamycin-treated mice. (C) Quantification of arterial re-endothelialization (blood vessel repair) using Evans Blue staining. Error bars denote ±SEM (n = 5), **p<0.01 using Student’s t-test. (D) Quantification of HUVEC migration across a growth factor gradient in the presence of either VEGF-A or basic FGF. Error bars denote ±SEM (n = 3), **p<0.01 using one-way ANOVA.

Does HSP90 inhibition perturb physiological responses such as blood vessel repair or endothelial cell migration? To test this, we used a mouse hind limb femoral artery injury model that evaluates endothelial regeneration after mechanical insult (Figure 6) [19], [30]. Control mice injected with vehicle alone showed a characteristic ‘injured’ arterial area denuded of endothelial cells with increased Evans Blue staining of the underlying extracellular matrix (Figure 6A). However, in mice subjected to treatment with the HSP90 inhibitor, a larger arterial area in the injured blood vessel was stained with Evans Blue suggesting that repair and regeneration of the arterial endothelium had been impaired (Figure 6B). Quantification showed a ∼2-fold reduction in arterial regeneration upon HSP90 inhibition (Figure 6C).

To assess the effects of HSP90 inhibition on endothelial cell migration, we used an assay where endothelial cells moved towards an increased concentration of pro-angiogenic growth factor i.e. VEGF-A or basic FGF. We titrated increasing HSP90 inhibitor concentrations under known concentrations of VEGF-A or basic FGF that stimulated primary endothelial cell migration (Figure 6D). There was a clear and preferential inhibition of VEGF-A-stimulated response inhibition in comparison to basic FGF stimulation in the presence of HSP90 inhibitor (Figure 6D).

Are HSP70 and HSP90 required for blood vessel development? To test this *in vivo*, morpholino-mediated knockdown of these HSPs were carried out in transgenic *Fli1-GFP* zebrafish embryos where the vascular endothelium expresses GFP. Confocal microscopy on the GFP-stained vasculature revealed significant defects in embryonic vascularization in the HSP70 and HSP90 morphants compared to control (Figure S3A). The dorsal longitudinal anastomotic vessels (DLAVs) of HSP70 and HSP90 morphants were not properly anastomosed (Figure S3A, red arrows), the intersegmental vessels (Se) were not appropriately orientated and spaced, and the tip cells of several Se split prematurely in two before reaching the dorsal side of the embryo (Figure S3A, white arrows). This gave a serrated appearance to the DLVAs compared to the control morphants. Moreover, in the caudal region of the HSP70 and HSP90 morphants, it was also difficult to distinguish the caudal vein and artery from the caudal vessel network (Figure S3A). Immunoblot analysis showed clear reduction in HSP70 (Figure S3B) or HSP90 (Figure S3C) levels in these experiments.

## Discussion

**Figure 7:**
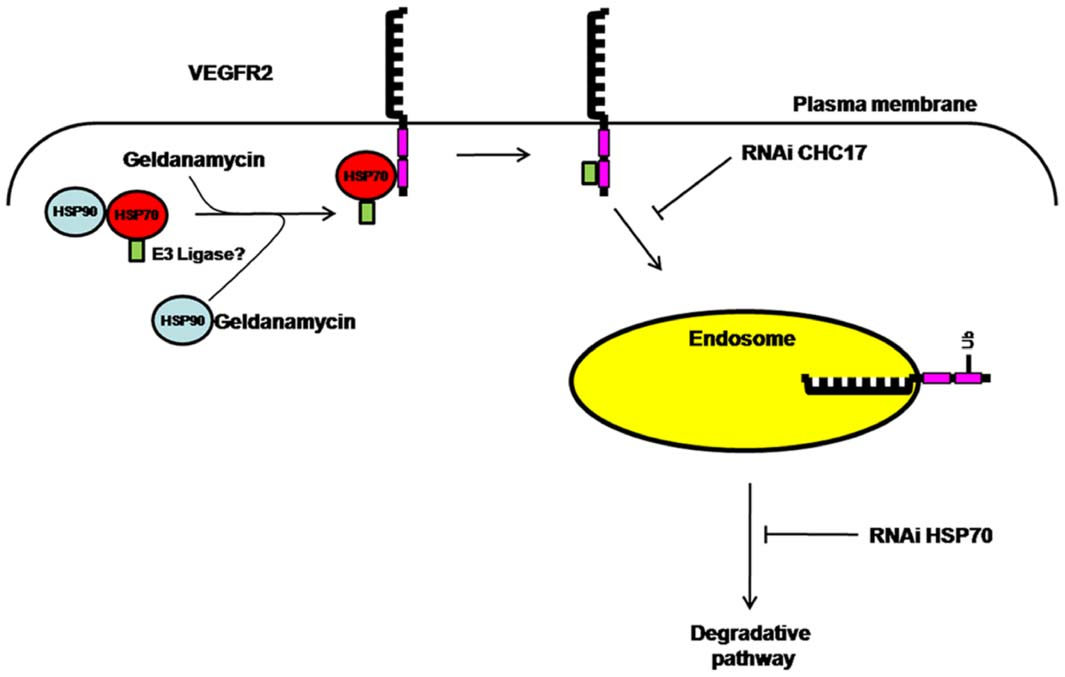
A model for HSP-mediated regulation of VEGFR2 proteolysis. Abbreviations: CHC17, clathrin heavy chain; E3 ligase, E3 ubiquitin ligase; HSP70, heat shock protein of 70 kDa; HSP90, heat-shock protein of 90 kDa, RNAi, RNA interference; Ub, mono/polyubiquitin modification(s); VEGFR2, vascular endothelial growth factor receptor 2.

VEGF-A regulates different aspects of vascular physiology in healthy and diseased states. Herein, we describe a novel mechanism involving the HSP70-HSP90 complex that targets VEGFR2 for degradation (Figure 7). The HSP90-specific pharmacological inhibitor, geldanamycin, triggered degradation of mature plasma membrane VEGFR2 in primary endothelial cells and caused a profound block in VEGF-A-stimulated intracellular signaling. Importantly, HSP90 inhibition stimulated increased VEGFR2-HSP70 complex formation that correlated with increased VEGFR2 degradation. Depletion of HSP70 could partially rescue this HSP-mediated degradative effect. The location of this HSP-regulated VEGFR2 degradation event is likely to occur in the endosome-lysosome system, as clathrin-mediated endocytosis is required. This HSP-mediated phenomenon was more specific for the VEGF-A-stimulated response, rather than the basic FGF-stimulated response evident by analysis of endothelial cell migration. Finally, there is a significant requirement for this HSP90-linked function in endothelial regeneration and blood vessel development, as HSP90 inhibition caused a 2-fold decrease in femoral artery repair in a mouse model and impaired vessel formation in transgenic zebrafish embryos.

What is the mechanism linking HSP activity to VEGF-A-regulated endothelial function? HSP90 and HSP70 are implicated in both protein stabilization and presentation to degradative pathways [7]. The uncoupling of a HSP70/HSP90 complex by the use of geldanamycin as an inhibitor of HSP90 activity could stimulate the recruitment of HSP70 to ‘client’ proteins linked to increased VEGFR2 ubiquitination and proteolysis by the 26S proteasome [31]. We thus postulate that inhibition of HSP90 activity stimulates HSP70 recruitment to VEGFR2 and increasess degradation by such a pathway. In this context, we have found increased VEGFR2 ubiquitination upon HSP90 inhibition (A.F.B., unpublished findings). Such a finding is consistent with postulation for recruitment of ubiquitin-linked machinery such an E3 ubiquitin ligase to the VEGFR2 cytoplasmic domain to mediate ubiquitin attachment followed by subsequent recognition by sorting machinery in the endosome and/or proteolysis by the cytosolic 26S proteasome (Figure 7). Multiple roles for HSP90 in endothelial function have been postulated [24], [26], [27], [32], [33] and various members of the VEGF-A-regulated signaling pathway in endothelial cells are HSP90 client proteins, including VEGFR2, Akt and eNOS [11], [23], [24], [26], [32], [34]. Importantly in the present study, pharmacological inhibition of HSP90 activity revealed increased HSP70 recruitment to a VEGFR2 complex but HSP90β binding was not evident. Whilst previous findings suggest a requirement for a HSC70/HSP70 complex in VEGF-A-mediated signaling [35] and a HSP70/HSP90 complex in pro-angiogenic endothelial responses [36], our results contradict studies showing that HSP90 directly binds to VEGFR2 [25], [37]–[39]: a HSP90/VEGFR2 complex exists in unstimulated endothelial cells [38], an interaction which is increased upon stimulation with VEGF-A and decreased by treatment with geldanamycin [25], [38]. Moreover, this interaction is required for assembly of a RhoA-ROCK complex, subsequent phosphorylation of focal adhesion kinase (FAK) [25], [37], [38] and recruitment of vinculin to VEGFR2 during focal adhesion assembly [38]. Another study reported class II histone deacetylases (HDACs) as co-ordinators of HSP-mediated VEGFR regulation; interestingly, treatment of cancer cell lines with a HDAC inhibitor decreased HSP90 binding, but increased HSP70 binding to VEGFR2 [39].

One explanation for the effects on VEGFR2 observed upon HSP90 inhibition and HSP70 recruitment is a partial unfolding of the cytoplasmic tyrosine kinase domain. For example, HSP90 inhibition can cause partial unfolding of the ErbB2 kinase domain followed by sequential recruitment of HSC70/HSP70 and E3 ubiquitin ligases such as Cullin5 or CHIP [28], [29], [40]. HSP70 depletion partly rescued VEGFR2 levels in HSP90-inhibited endothelial cells and overexpressed HSP70 co-localized with VEGFR2. Depletion of HSP70 only partly restored VEGFR2-mediated signaling. There are several possible explanations for this incomplete rescue besides the partial unfolding of the VEGFR2 tyrosine kinase domain: firstly, the knockdown of HSP70 is not complete. This may in part be due to a stress response in primary cells elicited by the foreign siRNA or transfection procedure, leading to up-regulation of heat shock proteins [41]–[43]. Secondly, treatment with geldanamycin could lead to enhanced internalization of VEGFR2 prior to conjugation onto HSP70 and consequently receptor degradation. Finally, combined inhibition of HSP90 and depletion of HSP70 may have a negative influence on the activity and/or expression of components of the VEGF-A-mediated signaling cascade, as both heat shock proteins have been purported to modulate multiple arms of this pathway [25], [35].

Our findings of increased ubiquitination within geldanamycin-treated VEGFR2 protein complexes (A.F.B, unpublished findings) suggest recruitment of a specific E3 ubiquitin ligase. Various E3 ligases have been postulated to mediate VEGFR2 ubiquitination including c-Cbl and βTrcP2 but this still remains contentious [13], [20], [21]. VEGFR2 also did not display association with the E3 ubiquitin ligase CHIP that is associated with HSP70-HSP90 complexes [5]. Thus it is likely there is an as yet unidentified E3 ubiquitin ligase that targets VEGFR2 (Figure 7). Clearly, E3 ubiquitin ligase activity can be co-ordinated by HSP90 activity to target another receptor tyrosine kinase, ErbB2 [40].

How does intracellular trafficking regulate VEGFR2 turnover? New biosynthetic VEGFR2 is transported relatively slowly through the secretory pathway to the plasma membrane. This mature VEGFR2 exhibits significant turnover (*t*_1/2_ ∼90–120 min) even in the absence of VEGF-A stimulation [13]. HSP90 inhibition promotes slow but sustained VEGFR2 degradation linked to endosomes (A.F.B., unpublished findings). Blocking receptor-mediated endocytosis via clathrin heavy chain (CHC17) depletion elevated VEGFR2 levels, indicating that plasma membrane internalization and delivery to the endosome-lysosome system precedes degradation. Ligand-stimulated and activated VEGFR2 also requires transport to early endosomes before proteasome-regulated removal of the cytosolic domain which modulates downstream intracellular signaling and endothelial cell migration [13]. VEGFR2 also undergoes endocytosis and recycling via early endosomes back to the plasma membrane [14], [44]. One likelihood is that HSP90 inhibition stimulates an existing endosome-linked pathway for proteolysis by the cytosolic 26S proteasome. Interestingly, HSP90 inhibition also stimulates proteolysis of the ErbB2 receptor tyrosine kinase associated with breast cancer tumor metastasis [4], [6]. In this context, ErbB2 endocytosis and localization to the endosome-lysosome pathway correlates with proteasome- and lysosome-mediated degradation [45]. Our model now identifies an existing route involving HSP90 and HSP70 in promoting receptor tyrosine kinase proteolysis in the endosome-lysosome pathway.

This HSP activity is indeed required for physiological responses such as endothelial cell migration and blood vessel repair. Firstly, VEGF-A-regulated cell migration is preferentially targeted in comparison to basic FGF in the presence of geldanamycin. Secondly, mouse femoral artery injury and regeneration was impaired ∼2-fold by geldanamycin, suggesting that re-endothelialization is dependent on a functional HSP70-HSP90 axis. In addition, HSP70 and HSP90 are also required for blood vessel development and sprouting in zebrafish (C.P.M. and P.F., unpublished findings). HSP90 inhibition by geldanamycin can block tumour angiogenesis [46], [47], possibly by lowering VEGF-A production by tumor cells, reducing expression of VEGFRs in endothelial and lymphatic tissue [11] or modulating expression of master signaling regulators Akt and c-Raf [10] and subsequent nitric oxide production [34], [48]. However, it has been difficult to assess the separate effects of this drug on tumor cell growth versus endothelial physiology [10], [25], [33], [46]–[49]. Here, we suggest that HSP90 inhibition could alter the endothelial response by stimulating VEGFR2 proteolysis, thus further validating the use of geldanamycin (and other HSP90 inhibitors) as agents to block tumor angiogenesis and highlighting differential pharmacological effects in vascular physiology versus tumor progression.

## Supporting Information

Figure S1 **HSP90 inhibition triggers VEGFR2 proteolysis and blocks VEGF-A-stimulated intracellular signaling.** (A) Radicicol treatment of endothelial cells also stimulates VEGFR2 degradation. VEGFR2 levels were detected using immunoblotting as previously shown. Immunoblot data were quantified and error bars denote ±SEM (n≥3), **p<0.01 using Student’s t-test. (B–C) HUVEC control (DMSO) or pre-treated with 1 µM geldanamycin (4 h) were combined with VEGF-A stimulation (5 min) followed by immunoblotting (IB) of whole cell lysates for intracellular signaling enzymes (B) phospho-pLCγ1 and (C) phospho-ERK1/2 (p42/44MAPK). Immunoblot data were quantified and error bars denote ±SEM (n≥3), ***p<0.005 using one-way ANOVA. (D) Transferrin receptor (TfR) levels are not perturbed by geldanamycin treatment. Cells treated with DMSO vehicle alone (control) or geldanamycin were subjected to immunoblotting as previously described and protein levels analyzed.
(TIF)

Figure S2 **Clathrin heavy chain CHC17 knockdown and effects on VEGFR2 trafficking and proteolysis.** (A) Knockdown of clathrin heavy chain CHC17 followed without or with geldanamycin and effects on VEGFR2 levels. Immunoblot data were quantified and error bars denote ±SEM (n≥3), **p<0.01 using one-way ANOVA. (B) Quantification of transferrin receptor (TfR) levels upon knockdown of clathrin heavy chain CHC17 without or with geldanamycin.
(TIF)

Figure S3 **Requirement for HSP70 or HSP90 in zebrafish blood vessel development.** (A) *Fli1-GFP* zebrafish embryos injected with control morpholinos or with morpholinos specific for HSP70 or HSP90β followed by vasculature staining for GFP. The results shown are representative of two experiments in each of which at least 100 embryos were injected with each morpholino. CA, Caudal Artery; CV, Caudal Vein; DLAV, Dorsal Longitudinal Anastomotic Vessel; Se, Intersegmental Vessel; DA, Dorsal Aorta; PCV, Posterior Cardinal Vein. (B) HSP70 or (C) HSP90β protein levels determined by immunoblot analysis; tubulin served as a control.
(TIF)
