# Supplementary figures and images for: A Heat-Shock Protein Axis Regulates VEGFR2 Proteolysis, Blood Vessel Development and Repair
Source: PLoS One. 2012 Nov 6;7(11):e48539. doi: 10.1371/journal.pone.0048539 (PMC3491040; doi:10.1371/journal.pone.0048539)

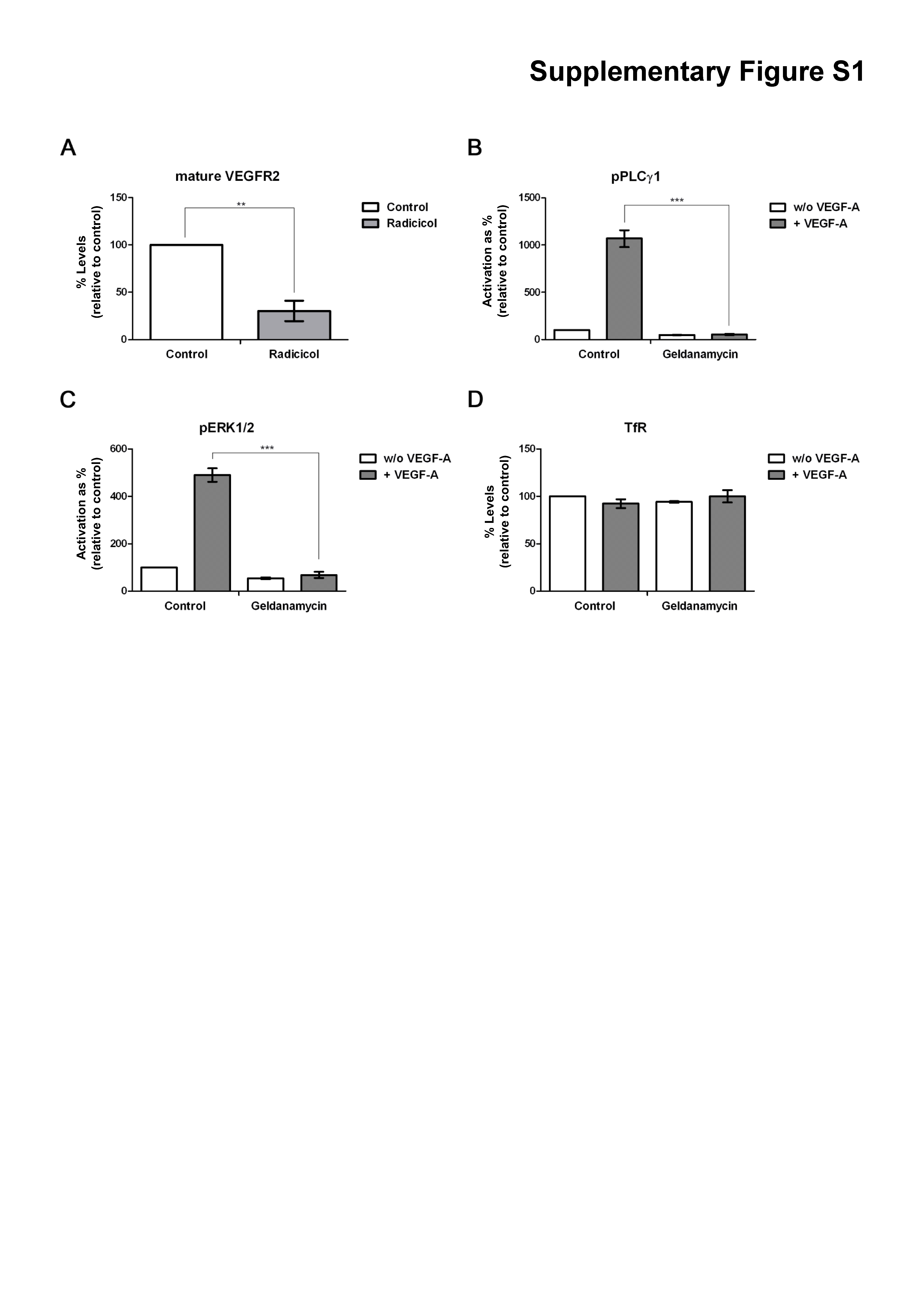

Supplement: Figure S1 — HSP90 inhibition triggers VEGFR2 proteolysis and blocks VEGF-A-stimulated intracellular signaling. (A) Radicicol treatment of endothelial cells also stimulates VEGFR2 degradation. VEGFR2 levels were detected using immunoblotting as previously shown. Immunoblot data were quantified and error bars denote ±SEM (n≥3), **p<0.01 using Student’s t-test. (B–C) HUVEC control (DMSO) or pre-treated with 1 µM geldanamycin (4 h) were combined with VEGF-A stimulation (5 min) followed by immunoblotting (IB) of whole cell lysates for intracellular signaling enzymes (B) phospho-pLCγ1 and (C) phospho-ERK1/2 (p42/44MAPK). Immunoblot data were quantified and error bars denote ±SEM (n≥3), ***p<0.005 using one-way ANOVA. (D) Transferrin receptor (TfR) levels are not perturbed by geldanamycin treatment. Cells treated with DMSO vehicle alone (control) or geldanamycin were subjected to immunoblotting as previously described and protein levels analyzed. (TIF) [file pone.0048539.s001.tif]

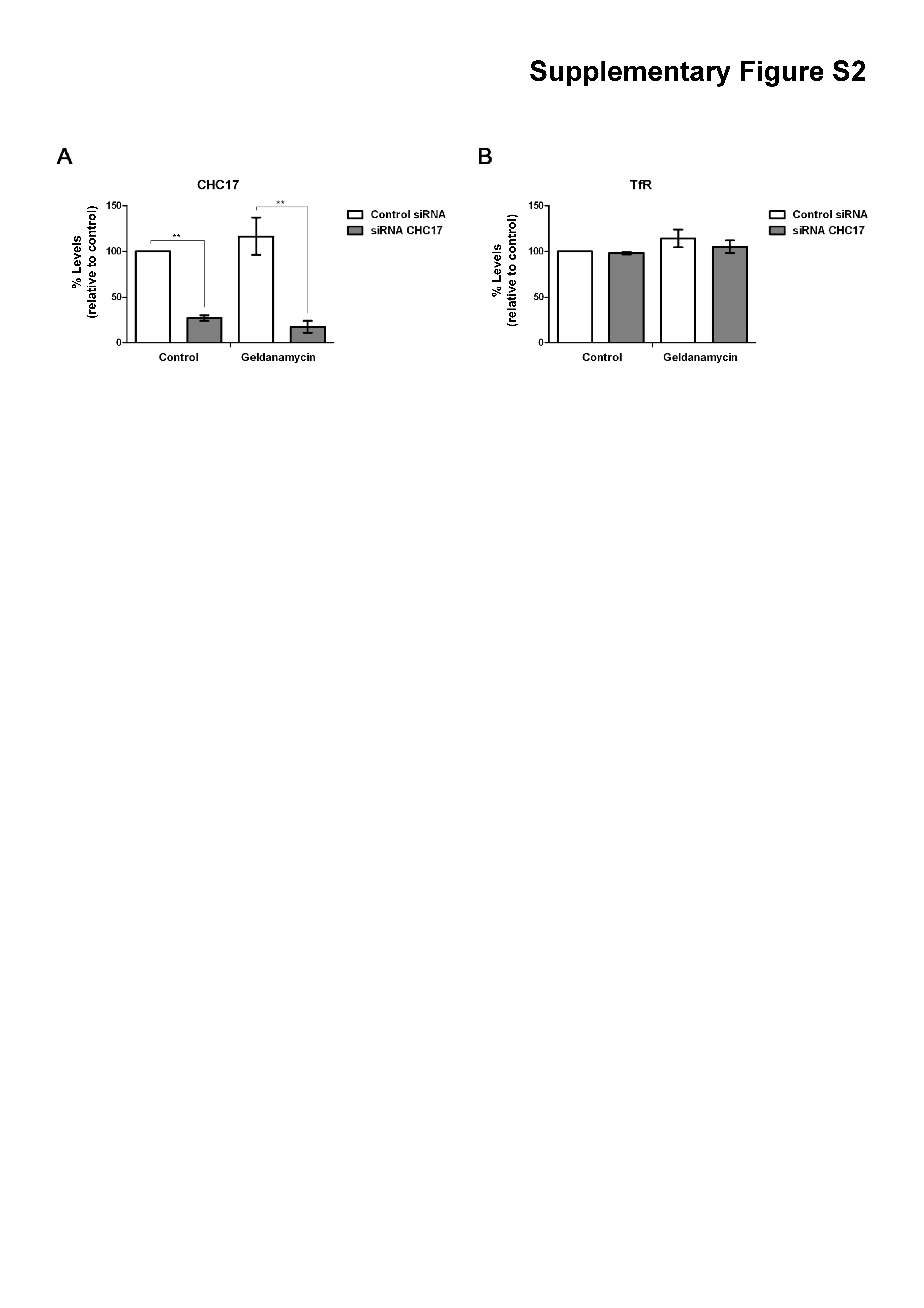

Supplement: Figure S2 — Clathrin heavy chain CHC17 knockdown and effects on VEGFR2 trafficking and proteolysis. (A) Knockdown of clathrin heavy chain CHC17 followed without or with geldanamycin and effects on VEGFR2 levels. Immunoblot data were quantified and error bars denote ±SEM (n≥3), **p<0.01 using one-way ANOVA. (B) Quantification of transferrin receptor (TfR) levels upon knockdown of clathrin heavy chain CHC17 without or with geldanamycin. (TIF) [file pone.0048539.s002.tif]

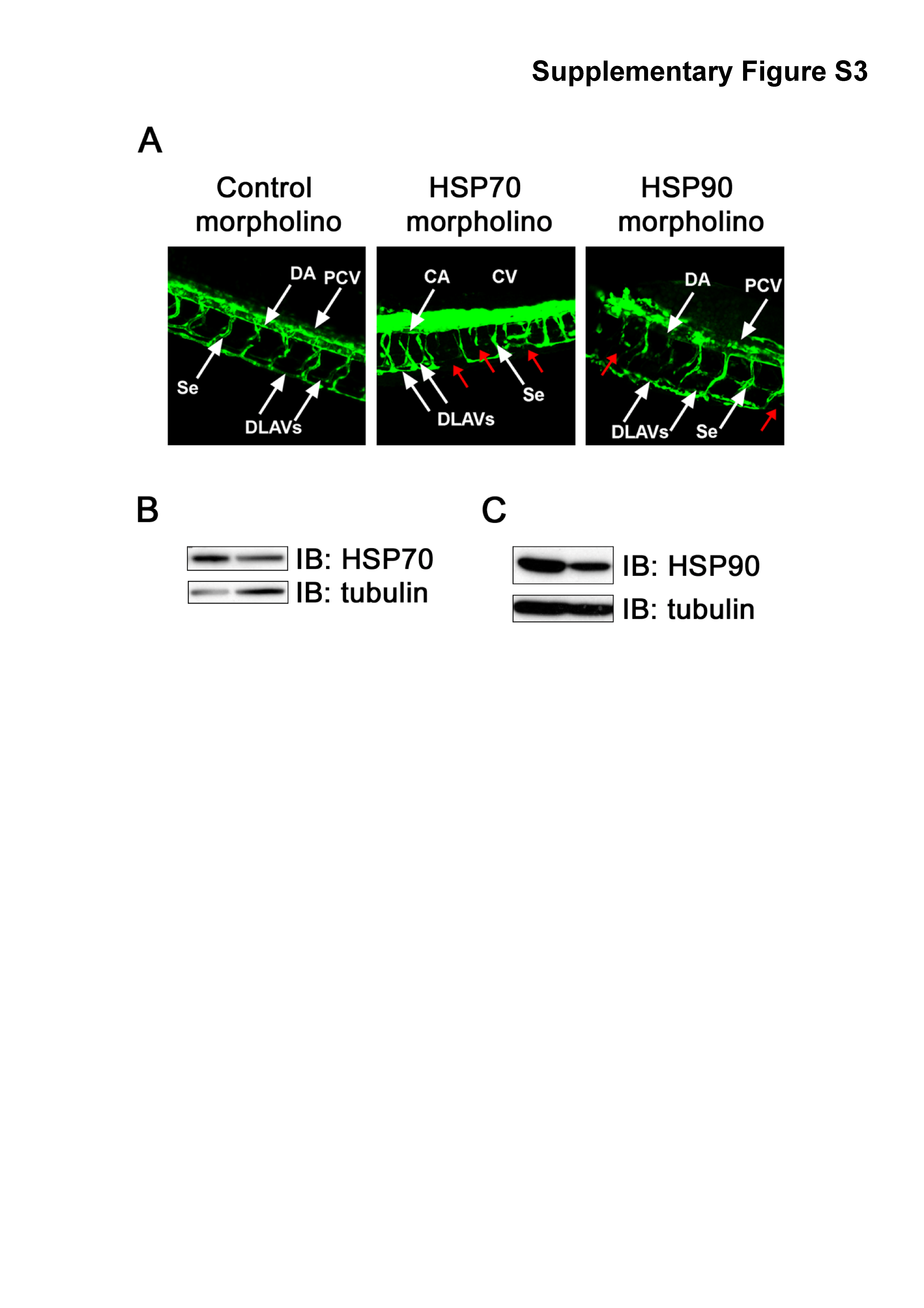

Supplement: Figure S3 — Requirement for HSP70 or HSP90 in zebrafish blood vessel development. (A) Fli1-GFP zebrafish embryos injected with control morpholinos or with morpholinos specific for HSP70 or HSP90β followed by vasculature staining for GFP. The results shown are representative of two experiments in each of which at least 100 embryos were injected with each morpholino. CA, Caudal Artery; CV, Caudal Vein; DLAV, Dorsal Longitudinal Anastomotic Vessel; Se, Intersegmental Vessel; DA, Dorsal Aorta; PCV, Posterior Cardinal Vein. (B) HSP70 or (C) HSP90β protein levels determined by immunoblot analysis; tubulin served as a control. (TIF) [file pone.0048539.s003.tif]
